# Serum level of soluble Interleukin-2 receptor among human papilloma virus infected female patients

**DOI:** 10.1186/s13027-025-00721-8

**Published:** 2025-12-17

**Authors:** Marwa M. Yasin, Rania A. Hassan, Abeer A. El Sayed, Sahar S. Ezzelarab, Mustafa M. Helal, Amira S. Abdelhady

**Affiliations:** 1 Medical Microbiology& Immunology Department, Faculty of Medicine, Ain Shams University, Cairo, Egypt; 2 Community Medicine Department, Faculty of Medicine, Ain Shams University, Cairo, Egypt; 3 Obstetrics & Gynecology Department, Faculty of Medicine, Ain Shams University, Cairo, Egypt

## Abstract

Progression from infection with human papillomavirus (HPV) to cervical cancer in some women is thought to involve a permissive host environment, one in which immune response is mobilized in an inappropriate manner. Interleukin-2 (IL-2) is one of the most studied cytokines driving T-cell proliferation and survival. Soluble Interleukin-2 receptor (sIL2R) was found to play an immunoregulatory role and is detected in the serum of healthy individuals but increases in association with certain types of neoplasms. The aim of the current study was to assess the serum level of sIL2R in HPV infected female patients with genital warts compared to healthy control women and to correlate the results with cervical cytology (pap smear) results to determine the utility of measuring the serum sIL2R levels in screening of precancerous changes in cervix. The study was conducted on 90 Egyptian female patients with genital warts and 90 apparently healthy controls. Cervical brush samples were taken for both conventional Pap cytology and detection of HPV-DNA. Serum levels of sIL2R were measured by enzyme-linked immunosorbent assay (ELISA). Patients were divided into two groups according to the result of Pap smear: group 1 with Low-grade squamous intraepithelial lesion (LSIL) having flat condyloma on cervix and group 2 with normal pap smear having condyloma accuminata lesions. Serum sIL2R levels of HPV infected patients showed a statistically significant higher median when compared to controls (p value < 0.0001). Also, group 1 showed a statistically significant higher median compared to group 2 regarding sIL2R levels (p value < 0.0001). sIL2R was found to be perfect and reliable in differentiation between group 1 and group 2. Also, it may be useful to use serum sIL2R level for screening of HPV infections and cervical cancer.

**Supplementary Information:**

The online version contains supplementary material available at 10.1186/s13027-025-00721-8.

## Introduction

Human papillomavirus infection has become one of the most common viral infections worldwide [1]. Persistent infection with HPV is the main cause of cancer cervix and other precancerous lesions as cervical intraepithelial neoplasia (CIN) [2].

There are more than forty types of HPV infecting the genital mucosa that can be divided into high risk (HR) HPV types and low risk (LR) HPV types [3], the HR types are associated with cervical precancerous lesions (such as CIN including flat condyloma of cervix) and cancer cervix, HPV-16 is the most prevalent HR HPV type, followed by HPV-18 [4, 5]. While the LR types are associated mainly with genital warts taking exophytic pattern of growth or condyloma accuminata which are caused mainly by types 6 and 11 [6].

Because of the disturbing nature of conventional modalities in treatment and a high rate of recurrence, immunotherapy has become a recent successful therapeutic modality for genital warts proven to have less side effects, lower recurrence rate and better outcome [6]. In addition, it gives better results than traditional therapies in patients with multiple lesions due to its systemic effect [7].

Interleukin-2 is one of the most studied cytokines driving T-cell proliferation and survival. IL-2 binds to the IL-2 receptor consisting of three separate chains [8]. The alpha subunit of IL-2 receptor that is released into the serum after shedding by proteolytic cleavage in association with T lymphocytes activation is called sIL2R [9].

It is now well known that sIL2R is an important immunological marker, the serum level of sIL2R is found to be increased in a variety of pathological conditions which include some types of cancer, autoimmune diseases and some inflammatory conditions [10], It has different clinical applications, as it may help to facilitate diagnosis of some immunological diseases, to monitor disease activity, severity and response to treatment and to predict benefits from specific therapeutic approaches [11] .

Several other interleukins, such as IL-6, IL-10, IL-12, and IL-17, have been implicated in the pathogenesis of HPV infection and cervical cancer which may play important roles in the tumor microenvironment. For instance, IL-6 promotes tumor cell proliferation and angiogenesis; IL-10 exerts immunosuppressive effects that favor viral persistence; IL-12 enhances Th1-mediated antitumor immunity; and IL-17 is involved in chronic inflammation and tumor promotion [12, 13]. However, IL-2 remains particularly significant due to its dual function, as it not only activates effector immune responses but also regulates immune tolerance through regulatory T cells (Tregs), maintaining a delicate balance between cellular immunity and tumor progression [14, 15].

Unlike many cytokines that act transiently or nonspecifically, IL-2 directly regulates T cell proliferation, differentiation, and cytotoxicity [8] which are key processes in clearing HPV infected keratinocytes. SIL2R, shed from activated lymphocytes, provides a stable systemic indicator of immune activation, enabling the distinction between transient and persistent HPV infections [16].

Furthermore, IL-2 has been the subject of numerous clinical trials in cancer immunotherapy, and recombinant IL-2 has already demonstrated efficacy in stimulating antitumor immunity in several malignancies, including melanoma, renal cell carcinoma and cervical cancer [17, 18]. These characteristics make IL-2 and its receptor system a promising model for understanding immune regulation in HPV-induced cervical carcinogenesis and for developing targeted immunotherapeutic strategies in gynecological oncology. Therefore, focusing on IL-2 and sIL-2R allows for both mechanistic insights and clinical relevance in understanding HPV-related cervical lesions.

Changes in serum level of sIL2R in patients with different types of HPV have been studied in previous studies seeking to understand the underlying cause of immunological imbalance in those patients [16, 19] and others considered sIL2R a useful marker for evaluating HPV disease stage [20]. This study aimed to assess the serum level of sIL2R in HPV infected female patients compared to healthy control women.

## Subjects and methods

This study was conducted on 90 Egyptian female patients with genital warts attending the early cancer detection unit at gynecology and obstetrics hospital in Ain-Shams University, and 90 apparently healthy age matched women served as a control group. Females having any immunologic or chronic disease were excluded from control group. This study was done during the period from May 2024 until December 2024.

The study protocol was reviewed and approved by the Research Ethical Committee, Faculty of Medicine, Ain-Shams University under approval number FWA000017585. All procedures performed in this research involving human participants complied with the ethical standards of the institutional research committee and with the 1964 Helsinki Declaration and its later amendments. Written informed consents were obtained from all participants after explanation of the aim and procedures of the study.

The following data were collected from patients and controls:

Marital status, number of marriages, duration of marriage, parity, age at menarche, age at first coitus, history of abortion, contraception, smoking and the presenting symptoms.

Patients were further divided into 2 groups according to the following parameters (Table 1):

**Table 1:** Parameters for division of study HPV infected patients into group 1 and group 2

	Group 1 HPV infected patients	Group 2 HPV infected patients
Description of warts clinically	Flat condyloma	Condyloma accuminata (exophytic papillary lesion)
Site of warts	Cervix	Vulva and vagina
Result of Pap smear according to 2014 Bethesda System For Reporting Cervical Cytology [21]	Low-grade squamous intraepithelial lesion (LSIL) encompassing mild dysplasia/CIN 1	Normal: Negative for Intraepithelial Lesion or Malignancy (NIL)

### Specimen collection and processing

#### Cervical brush samples

Cervical cell scrapings were obtained by a gynecologist using a sterile cytobrush. the procedure was clearly explained to each patient and informed consent was obtained prior to sample collection. The patient was positioned in the lithotomy position and a sterile Cusco speculum was gently inserted into the vagina to visualize the cervix. Excess cervical and vaginal secretions were carefully wiped off. A cervical cytobrush was then inserted into the endocervical canal, gently rotated 3 to 5 times to collect an adequate sample of cervical epithelial cells [21]. The collected material from cytobrush was immediately spread thinly and evenly onto a clean glass slide. The slide was then fixed immediately by immersion in 95% ethyl alcohol to prevent air-drying and cellular distortion [22]. The cytobrush was then placed in RPMI specimen transport medium (GIBCO diagnostics, USA), and transported to the lab. Samples were stored at -80 °C until processed for detection of HPV-DNA [23].

#### Blood samples

2-3 ml venous blood were obtained from all the participants by venipuncture using aseptic technique. The samples were transferred into serum separation tube; left to clot for 30 min., then centrifuged at 3000 rpm for 15 min. Serum was separated and then stored at -80 °C for further processing [24].

### Cytological examination

Cytological examination was performed at the pathology lab and findings were classified according to the 2014 Bethesda classification system [25].

### Detection of HPV-DNA by polymerase chain reaction (PCR)

The 90 female patients were confirmed to have HPV infection by HPV conventional PCR testing. DNA extraction was done using thermo scientific GeneJET Genomic DNA Purification Kit (catalog no. K0721) for purification of DNA from cervical samples according to manufacturer’s instructions.

Amplification of a 450 bp segment of HPV-DNA was carried out using the Forward primer (MY09): 5`-CGT CCA AAA GGA AAC TGA GC-3` and the Reverse primer (MY11): 5`-GCA CAG GGA CAT AAC AAT GG-3` (Invitrogen, USA) [26]. In addition, all samples were examined for DNA integrity by amplification of the β-globin gene as internal control using the Forward primer (PC04): 5’ CAA CTT CAT CCA CGT TCA CC 3’ and the Reverse Primer (GH20): 5’ GAA GAG CCA AGG ACA GGT AC 3’. The resultant product was expected to be a 250-bp fragment [27]. Each run included a proven HPV positive sample as positive control and a negative control. Amplification was performed with the following cycling profile according to Sotlar et al. [26]: 1 cycle of initial denaturation at 95 °C for 2 min followed by 40 cycles of denaturation at 95 °C for 45 s, annealing at 55 °C for 45 s, extension at 72 °C for 60 s followed by 1 cycle of final extension at 72 °C for 2 min using Thermo Electron Corporation thermal cycler (USA) .The amplified products were detected by gel electrophoresis on 2% agarose gel and visualized using ethidium bromide [27].

### Assessment of serum sIL2R level using ELISA

Serum sIL2R concentrations were measured using commercial quantitative double antibody sandwich ELISA kits (Bioassay Technology Laboratory, Shanghai, China) following the manufacturer’s instructions.

### Statistical analysis

The collected data were revised, coded, tabulated and introduced to a PC using statistical package for social sciences (IBM SPSS 20.0). Data were presented and suitable analysis was done according to the type of data obtained for each parameter. Descriptive Statistics was expressed as Mean, standard deviation (± SD) and range for parametric numerical data, while median and interquartile range (IQR) for nonparametric data. Analytical Statistics was done by Chi square test or fisher’s exact test.

## Results

### Demographic and reproductive data

Demographic and reproductive characters of patients and control groups are presented in Table 2.

**Table 2:** Demographic and reproductive characteristics of both HPV infected patients and control groups

		Group		Test of significance		
		Healthy controls (*n* = 90)	HPV infected patients (*n* = 90)			
		Mean ± SD *N* (%) Median (IQR)	Mean ± SD *N* (%) Median (IQR)	value	*p*-value	Sig.
Age		36.67 ± 5.27	36.7 ± 5.41	***t=*** -0.042	0.967	NS
Number of marriages	1	90 (100%)	63 (70%)	FE	< 0.001	S
	2	0 (0%)	15 (16.67%)			
	3	0 (0%)	6 (6.67%)			
	4	0 (0%)	6 (6.67%)			
Duration of marriage (Years)		7 (3–8)	15 (10–18)	***z=*** -7.913	< 0.001	S
Parity		2 (1–3)	3 (2–4)	***z=*** -4.317	< 0.001	S
Abortus		0 (0–0)	0 (0–1)	***z=*** -4.99	< 0.001	S
Age at menarche (Years)		12.08 ± 0.43	12.03 ± 0.41	***t = *** 0.71	0.478	NS
Age at first coitus (Years)		24.73 ± 1.3	22.67 ± 3.59	***t = *** 5.135	< 0.001	S
Contraception	No	12 (13.33%)	6 (6.67%)	***X***^***2***^ ***=*** 10.769	0.005	S
	OC	48 (53.33%)	69 (76.67%)			
	Loop	30 (33.33%)	15 (16.67%)			
Pregnancy	No	78 (86.67%)	84 (93.33%)	***X***^***2***^ ***=*** 2.222	0.136	NS
	Yes	12 (13.33%)	6 (6.67%)			
Smoking	No	90 (100%)	90 (100%)			

*Student t-test of significance (t)

*Fisher’s Exact test of significance (FE)

*Mann-Whitney test of significance (z)

*Chi-Square test of significance (X^2^)

### Assessment of serum sIL2R level using ELISA

**Fig. 1:**
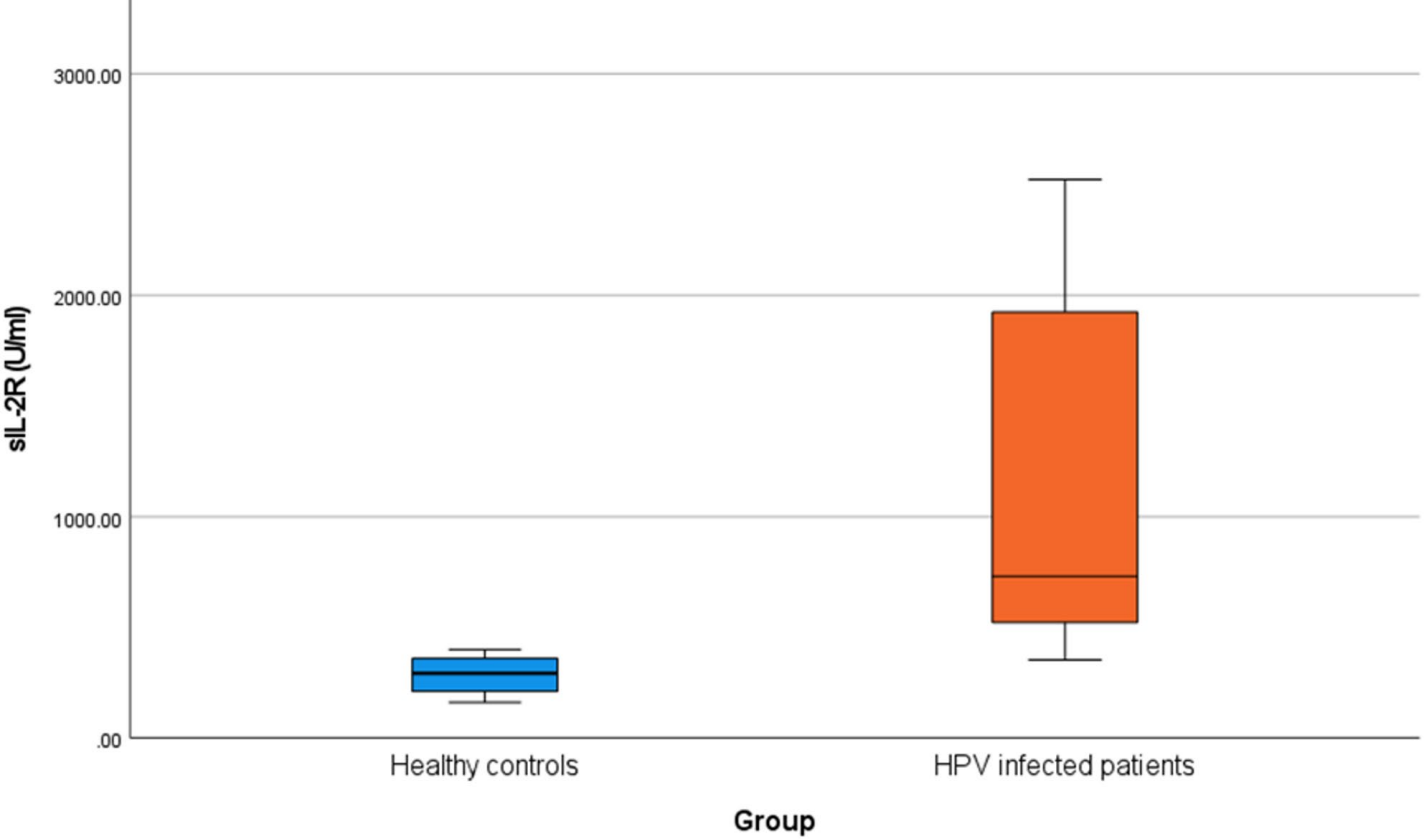
Difference in sIL-2R (U/ml) between HPV infected patients and healthy controls, HPV infected patients show a highly significant statistical elevation in sIL2R serum level compared to controls (p value < 0.001)

As regards the serum levels of sIL2R there was a highly significant statistical difference between HPV infected patients and control groups (p value < 0.001) (Table 3; Fig. 1).

**Table 3:** Comparison between HPV infected patients and control groups regarding sIL2R serum level

		Group		Mann-Whitney test		
		HPV infected patients (*n* = 90)	Healthy controls (*n* = 90)	***z***	p-value	Sig.
sIL-2R (U/ml)	Median (IQR)	731.5 (523–1924)	293 (211–360)	-11.177	< 0.001	HS
	Range	(353–2523)	(161–399)			

**Fig. 2:**
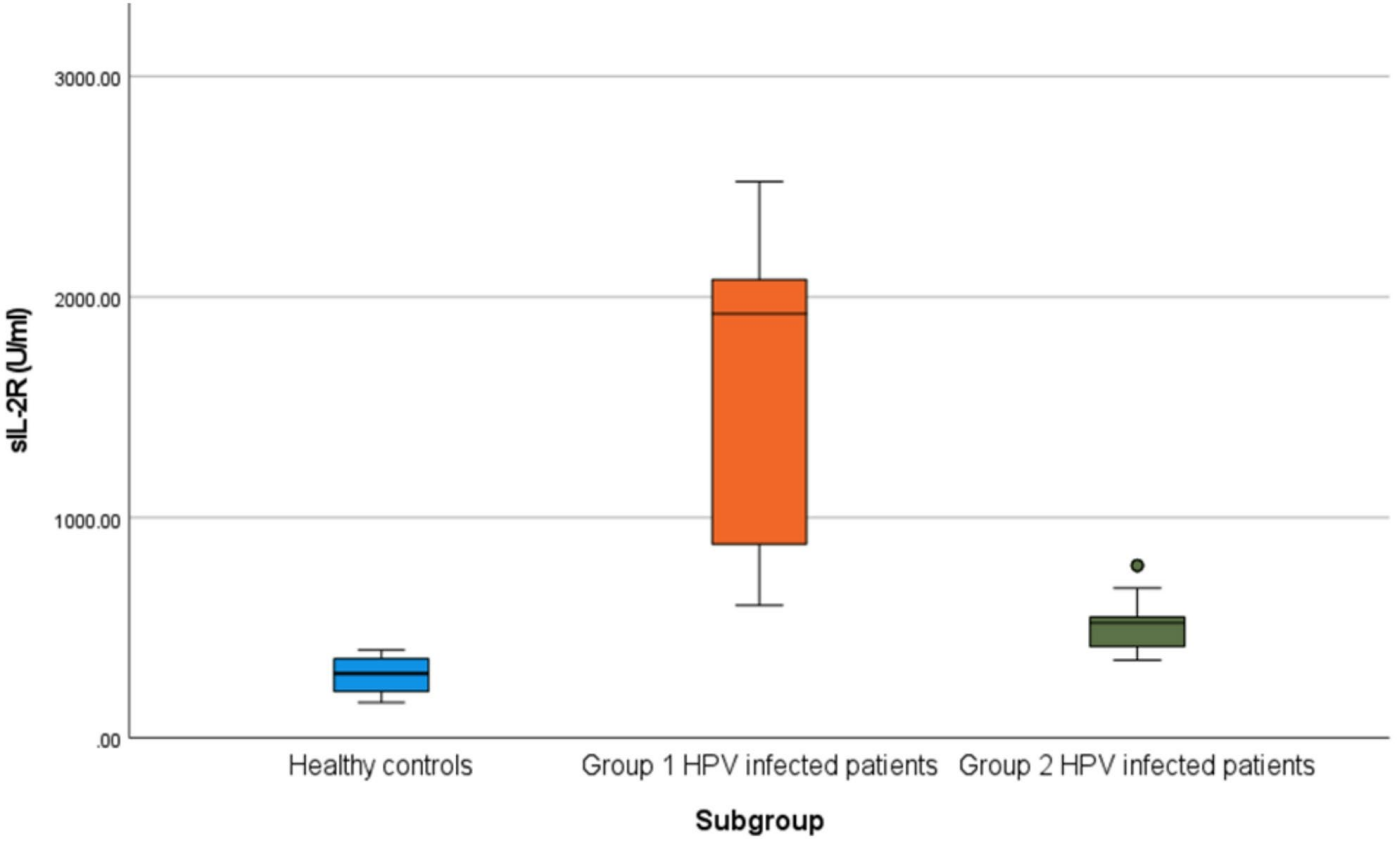
Difference in sIL-2R (U/ml) between three study groups, patients’ group 1 show a highly significant statistical elevation in sIL2R serum level compared to patients’ group 2 and to controls (p value < 0.001)

Results showed also that patients’ group 1 (patients with flat condyloma in cervix) showed a statistically highly significant higher serum sIL2R level when compared to controls (p value < 0.0001) as shown in Table 4. Also, patients’ group 2 (patients with condyloma accuminata) showed a statistically highly significant higher serum sIL2R level when compared to controls (p value < 0.0001) as shown in Table 5. In addition, there was highly statistically significant difference between both patients’ groups 1 and 2 as regards the level of sIL2R (p value < 0.0001), where group 1 had a higher median when compared to group 2 as shown in Table 6. These results are shown also in (Fig.2).

**Table 4:** Evaluation of sIL-2R between healthy controls and group 1 HPV infected patients

		Subgroup		Mann-Whitney test		
		Group 1 HPV infected patients (*n* = 45)	Healthy controls (*n* = 90)	***z***	p-value	Sig.
sIL-2R (U/ml)	Median (IQR)	1924 (880–2079)	293 (211–360)	-9.456	< 0.001	S
	Range	(602–2523)	(161–399)			

**Table 5:** Evaluation of sIL-2R between healthy controls and group 2 HPV infected patients

		Subgroup		Mann-Whitney test		
		Group 2 HPV infected patients (*n* = 45)	Healthy controls (*n* = 90)	***z***	p-value	Sig.
sIL-2R (U/ml)	Median (IQR)	523 (415–549)	293 (211–360)	-8.783	< 0.001	S
	Range	(353–783)	(161–399)			

**Table 6:** Evaluation of sIL-2R between group 1 and group 2 of HPV infected patients

		Subgroup		Mann-Whitney test		
		Group 1 HPV infected patients (*n* = 45)	Group 2 HPV infected patients (*n* = 45)	***z***	p-value	Sig.
sIL-2R (U/ml)	Median (IQR)	1924 (880–2079)	523 (415–549)	-8.03	< 0.001	S
	Range	(602–2523)	(353–783)			

**Fig. 3:**
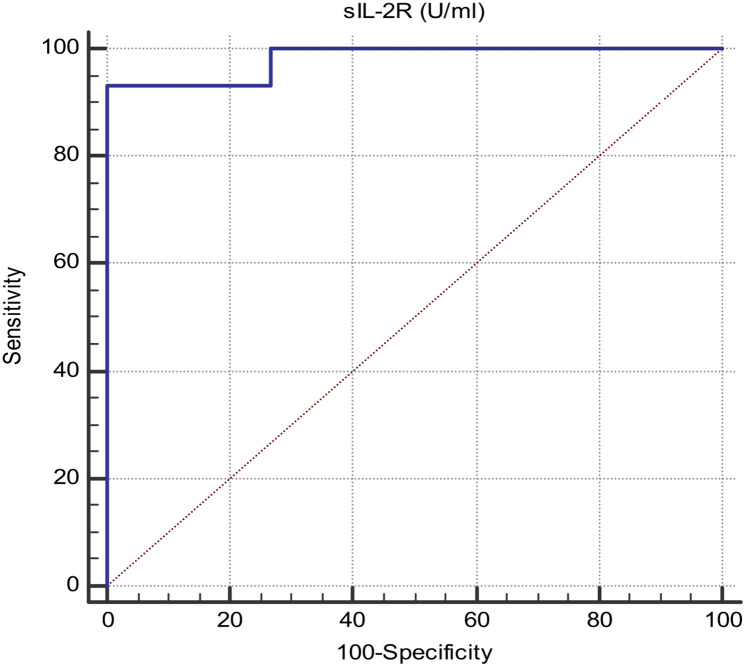
ROC curve of SIL2R to discriminate between HPV infected patients and healthy controls, Area under the curve (AUC) is 0.982, sensitivity 93.33%, specificity 100%, 95% CI from 0.967 to 0.997, p-value < 0.001 and cutoff value is 399 U/ml

Receiver operating characteristic curves were constructed which found that serum sIL2R level was perfect and reliable to predict HPV infection with a sensitivity of 93.33%, specificity 100%, p-value < 0.001 and the best cutoff value for sIL2R was 399 U/ml. (Fig. 3; Table 7).

**Table 7:** Diagnostic performance of sIL-2R to discriminate between HPV infected patients and healthy controls

	AUC	95% Confidence Interval (CI)	*p*-value	Cutoff value	Sensitivity	Specificity
sIL-2R (U/ml)	0.982	0.967 to 0.997	< 0.001	> 399	93.33	100

**Fig. 4:**
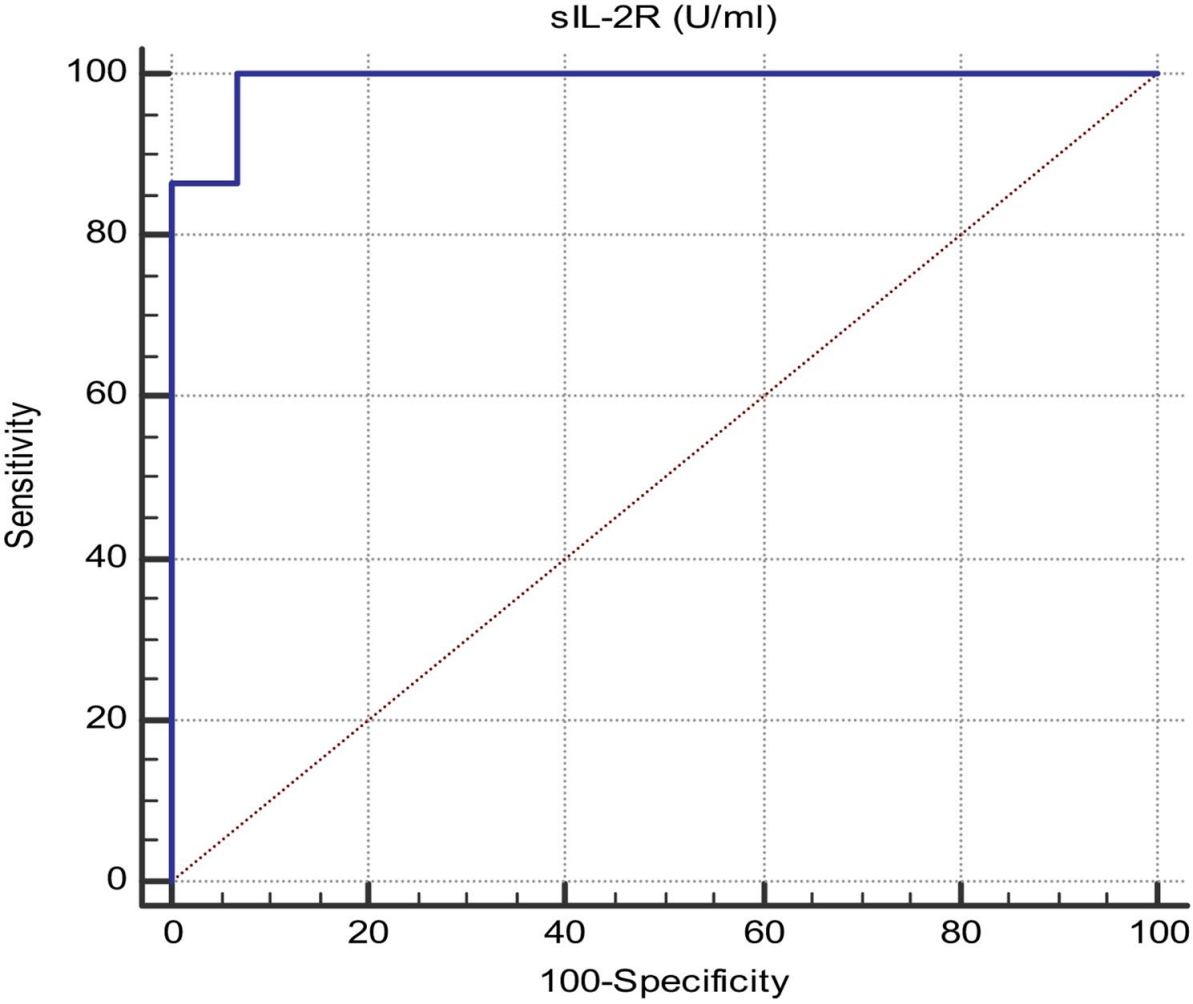
ROC curve of SIL2R to discriminate between Group 1 and Group 2 HPV infected patients: Area under the curve (AUC)is 0.991, sensitivity 100%, specificity 93.33%, 95% CI from 0.979 to 1.00, p-value < 0.001 and cutoff value is 783U/ml

Also, sIL2R level was perfect and reliable to differentiate between precancerous flat condyloma of cervix caused by HPV (group 1) and benign condyloma accuminata lesions of vulva and vagina (group 2) with a sensitivity 100%, specificity 93.33%, p-value < 0.001 and the best cutoff value sIL2R was 783U/ml. (Fig. 4; Table 8).

**Table 8:** Diagnostic performance of sIL-2R to discriminate between group 1 and group 2 HPV infected patients

	AUC	95% Confidence Interval (CI)	*p*-value	Cutoff value	Sensitivity	Specificity
sIL-2R (U/ml)	0.991	0.979 to 1.00	< 0.001	≤ 783	100	93.33

## Discussion

HPV infection has become one of the most considerable major health problems worldwide particularly among females causing around 90% of cancer cervix cases [28]. Also, it is the leading cause of genital warts which is the most common sexually transmitted disease [29]. So, it was important to understand the underlying immunological dysregulation in those patients. As sIL2R plays an immunoregulatory role [30], it was used to help in understanding this immunological dysregulation in HPV patients.

This study showed that increased number of marriages, and young age at first coitus are two risk factors for HPV infection. This result goes in accordance with the results reported by both Oyouni [3] and Baudu et al. [31], who stated that increased number of marriages and early age at first sexual intercourse are recognized as significant behavioral risk factors for HPV infection, as both are associated with increased lifetime sexual exposure and vulnerability to persistent infection. Also, Nunez et al. [32] reported that first sexual intercourse before 20 years of age is a high-risk factor for preinvasive and invasive lesions of the cervix.

In this study it was found that sIL2R was significantly higher in HPV infected patients than controls (p value < 0.0001), Elevated serum levels of sIL-2R represents sustained T-cell stimulation. During persistent human HPV infection, continuous exposure to viral antigens promotes prolonged activation of CD4⁺ and CD8⁺ T lymphocytes. Activated T cells express high levels of membrane-bound IL-2Rα (CD25), which can be cleaved and released into circulation as sIL-2R [33]. This elevated sIL-2R not only mirrors the intensity of immune activation but also indicates immune dysregulation, as chronic stimulation may exhaust T-cell responses, alter cytokine signaling, and reduce the efficiency of viral clearance [10]. IN case of persistent HPV infection, such immune exhaustion and regulatory imbalance may contribute to the maintenance of viral persistence and may facilitate the progression of HPV-related lesions toward cervical intraepithelial neoplasia or malignancy.

ROC curves revealed that sIL2R is perfect and reliable biomarker to predict HPV infection with an excellent diagnostic performance with a sensitivity of 93.33%, specificity up to 100%. This agreed with the result from Nayki et al. [16]. Results also revealed that sIL2R was significantly higher in HPV infected patients’ group 1 than group 2. ROC curves analysis indicated that sIL2R is a promising biomarker to predict precancerous changes in cervix with a sensitivity up to 100%, specificity 93.33% which indicates its potential role not only in diagnosis but also in screening. These results agreed with the results from Sha et al. [34] which revealed that serum sIL2R have higher diagnostic efficiency for cervical cancer as in ROC curve found that AUC of sIL2R was 0.813, the diagnostic sensitivity was 80.13%, and the specificity was 69.97%. These results were also consistent with those from Nayki et al. [16] and Arioz et al. [19] which showed that sIL2R significantly increased in patients with high-risk HPV infections.

The above-mentioned results suggest that the changes in sIL2R serum level in patients with premalignant lesions can predict the onset of cervical cancer to a certain extent. It is needful to find new noninvasive screening tools for precancerous and cancerous changes in cervix as cytology-based screening that has taken the upper hand in cervical cancer screening programs for a long period has some challenges such as low sensitivity ranging from 18.6% to 76.7% as well as relatively short screening intervals [35].

The results of the present study also showed that sIL2R was significantly higher in HPV infected patients group 2 having condyloma accuminata than healthy controls (P value < 0.0001), this can be a principal mechanism of the immune dysregulation in those patients, as high serum level of sIL2R causes apoptosis of effector T cells as a result of their deprivation of the growth factor IL-2, as sIL2R (CD25) is released as a decoy receptor to block free IL-2 from binding to effector T cells [36]. These results also can be a good explanation for the effective role of current immunotherapy as Mycobacterium vaccine in treatment of recurrent warts which works by stimulating T helper 1 cytokines such as IL2 as discussed by Thappa and Chiramel [37]. This also opens the way for new immunotherapy modalities such as systemic use of low dose of IL2.Therabuticallly, IL-2 has also been explored in cervical cancer immunotherapy models where it enhanced engineered T-cell efficacy [38].

This study, however, have some limitations. First, the sample size was relatively small, and the study population was homogenous and selected from single center which may limit the generalizability of the finding thus these results have to be validated in larger multicenter studies involving more diverse patient groups. In addition, sIL2R level were only assessed in serum while evaluating it in cervical tissue may give more stable results. Also, the control group was recruited based on the absence of genital lesions clinically and negative gynecologic examination, but HPV molecular testing was not performed because of scarcity of resources. The lack of molecular HPV testing in the control group may have affected the interpretation of serum sIL2R levels.

In conclusion, sIL-2R may serve as a promising biomarker for immune monitoring in HPV-infected individuals. Measuring sIL-2R levels can provide insight into the host immune activation state and help distinguish between transient and persistent infections. Elevated concentrations might also predict disease progression, identifying patients at greater risk for developing cervical precancerous lesions or cancer. Integrating sIL-2R assessment with other immunologic and virologic markers could improve the accuracy of disease staging and patient management. In addition, this opens the way for new treatment modalities for HPV patients as using IL-2 immunotherapy, this can participate in repairing such immunological imbalance to some extent and improve the outcome. Finally, Studies with a larger sample size are necessary to explain more clearly the relationship between sIL2R and cervical cancer.

## Supplementary Information

Below is the link to the electronic supplementary material.

Supplementary Material 1

## Data Availability

The datasets analyzed during the current study are not publicly available due to ethics in human research but are available from the corresponding author on reasonable request.
