# Supplementary material for: Serum level of soluble Interleukin-2 receptor among human papilloma virus infected female patients
Source: Infect Agent Cancer. 2025 Dec 17;21:7. doi: 10.1186/s13027-025-00721-8 (PMC12821268; doi:10.1186/s13027-025-00721-8)
Supplement: Supplementary file 1 — Supplementary Material 1 [file 13027_2025_721_MOESM1_ESM.docx]

**Author contribuation**

MMY and MMH assisted in the collection of samples and patients’ data. MMY and AS contributed to laboratory work and analysis of data. SS contributed to statical analysis of data . RA ,AA and AS assisted in manuscript drafting and revision. All authors contributed significantly to the study’s conception, design, and final approval of the manuscript.
